# Spatio-temporal activation patterns of neuronal population evoked by optostimulation and the comparison to electrical microstimulation

**DOI:** 10.1038/s41598-023-39808-w

**Published:** 2023-08-04

**Authors:** Shany Nivinsky Margalit, Hamutal Slovin

**Affiliations:** The Gonda Multidisciplinary Brain Research Center, Bar-Ilan University, Ramat Gan, Israel

**Keywords:** Neuroscience, Somatosensory system

## Abstract

Optostimulation and electrical microstimulation are well-established techniques that enable to artificially stimulate the brain. While the activation patterns evoked by microstimulation in cortical network are well characterized, much less is known for optostimulation. Specifically, the activation maps of neuronal population at the membrane potential level and direct measurements of these maps were barely reported. In addition, only a few studies compared the activation patterns evoked by microstimulation and optostimulation. In this study we addressed these issues by applying optostimulation in the barrel cortex of anesthetized rats after a short (Short_Exp_) or a long (Long_Exp_) opsin expression time and compared it to microstimulation. We measured the membrane potential of neuronal populations at high spatial (meso-scale) and temporal resolution using voltage-sensitive dye imaging. Longer optostimulation pulses evoked higher neural responses spreading over larger region relative to short pulses. Interestingly, similar optostimulation pulses evoked stronger and more prolonged population response in the Long_Exp_ vs. the Short_Exp_ condition. Finally, the spatial activation patterns evoked in the Long_Exp_ condition showed an intermediate state, with higher resemblance to the microstimulation at the stimulation site. Therefore, short microstimulation and optostimulation can induce wide spread activation, however the effects of optostimulation depend on the opsin expression time.

## Introduction

Electrical stimulation of the brain is a well-established technique that enables to artificially activate the brain tissue, evoke motor action and affect behavioral output. In addition, it can influence sensory perception and bias perceptually guided decisions^1–3^. Recent studies have demonstrated that electrical stimulation is an effective tool to generate artificial sensation and movements in rodents^4–6^ and in higher mammals^7–9^ (for review see^3,10,11^). Despite these important contributions, over recent years, the research focus has shifted to optogenetics and optostimulation techniques, where neuronal activity is controlled through light sensitive channels. Optostimulation using optogenetic tools, enables to overcome some of the major disadvantages of electrical stimulation. While electrical stimulation activates neuronal elements in an unspecific manner, in particular fibers and axons^12^, optostimulation gain a selective control of specific subpopulations of neurons via genetic markers^13^, and activates mainly cell bodies at the injection site^14^. Moreover, the optogenetic technique enables to induce depolarization or hyperpolarization at the targeted neurons by the choice of the opsin type^15,16^. Multiple studies showed that optogenetics can also successfully modulate behavior in rodents^17–19^ and recently also in higher mammals such as non-human primates^9^.

The effects of electrical stimulation on cortical neurons and neuronal population were extensively investigated and characterized^10,20,21^. Using voltage-sensitive dye imaging (VSDI), a recent study directly measured the spatio-temporal activation patterns of the neural responses to microstimulation^22^. This study reported that a short microstimulation pulse in the barrel cortex of anesthetized rats evoked increased neural activation that propagated horizontally within the barrel cortex and lasted much longer than the stimulation duration. Using an array of microelectrodes in the barrel cortex of anesthetized rats, Butovas et al.^23^ reported on large spatial spread responses to microstimulation, even at threshold intensity. Yet, the characteristics of cortical population response to short pulses of optostimulation was much less investigated, in particular the evoked spatio-temporal patterns of neuronal population spanning the full membrane potential range: sub-threshold and supra-threshold levels.

In addition, while both brain stimulation techniques are widely used in research, only a few studies compared the activation patterns of intracortical microstimulation (ICMS) and optostimulation. Millard et al.^24^ compared the differences between sensory stimulation of the facial vibrissae in anesthetized rats to electrical stimulation and optostimulation. They stimulated thalamic neurons projecting to layer 4 in the primary somatosensory cortex (S1) while imaging the population response in S1 using VSDI. They reported that electrical microstimulation of the thalamic neurons evoked a highly unnatural spatial activation in the barrel cortex, whereas optostimulation of the thalamic neurons evoked a cortical response that showed similar spatial properties to that induced by sensory vibrissa stimulation. Another study^25^ attempted to investigate both stimulation techniques by comparing the activation of koniocellular projections from the lateral geniculate nucleus (LGN) to primary visual cortex (V1) in the macaque monkey. Contrary to the previous study, they found that electrical microstimulation generated the same V1 activation pattern as the one elicited by optostimulation. However, the first study recorded the neuronal responses at 3–4 weeks after opsin injection while the last one performed the experiments at longer time after viral injection (up to 5 months). This raises a question whether the results of optostimulation on neuronal population can generate different patterns of activation, depending on the time elapsing from viral injection i.e. the expression time of the excitable channel?

To address the above issues, we used VSDI to measure and characterize the neural population response to short pulses of optostimulation in the barrel cortex of anesthetized rats. Optostimulation was applied at two different expression periods: 3–4 weeks (Short_Exp_) or 8 weeks (Long_Exp_) after AAV-ChR2 transfection in pyramidal neurons. Our results show that optostimulation generated cortical activation that spread few mm from the stimulation site. We found that optostimulation in the Short_Exp_ condition showed a smaller spread of activation and a faster decay in comparison with Long_Exp_. Next, we compared the temporal and spatial patterns of the neuronal population response induced by the optostimulation and microstimulation. The cortical response evoked by optostimulation applied in Long_Exp_, i.e. after longer ChR2 expression, showed higher resemblance to the spatio-temporal pattern of population response evoked by microstimulation. Therefore, both ICMS and optostimulation can induce wide spread neuronal response even to short stimulation, while the effects of optostimulation depend on the time elapsing from viral injection, that is the expression duration.

## Results

In this study, we first asked what are the spatio-temporal pattern of neuronal population response evoked by brief pulses of optostimulation. We investigated this for two different time windows of viral expression. We then compared these responses to those generated by electrical microstimulation. To study these topics, we measured the neuronal population activity using VSDI, at high spatial (mesoscale, 50^2^ µm^2^/pixel) and temporal resolution (100 Hz; see "Methods"). The fluorescence dye signal of each pixel reflects the sum of membrane potential from all neuronal elements in the pixel area and emphasizes subthreshold potential but reflects also suprathreshold membrane potentials^26–28^.

**Figure 1:**
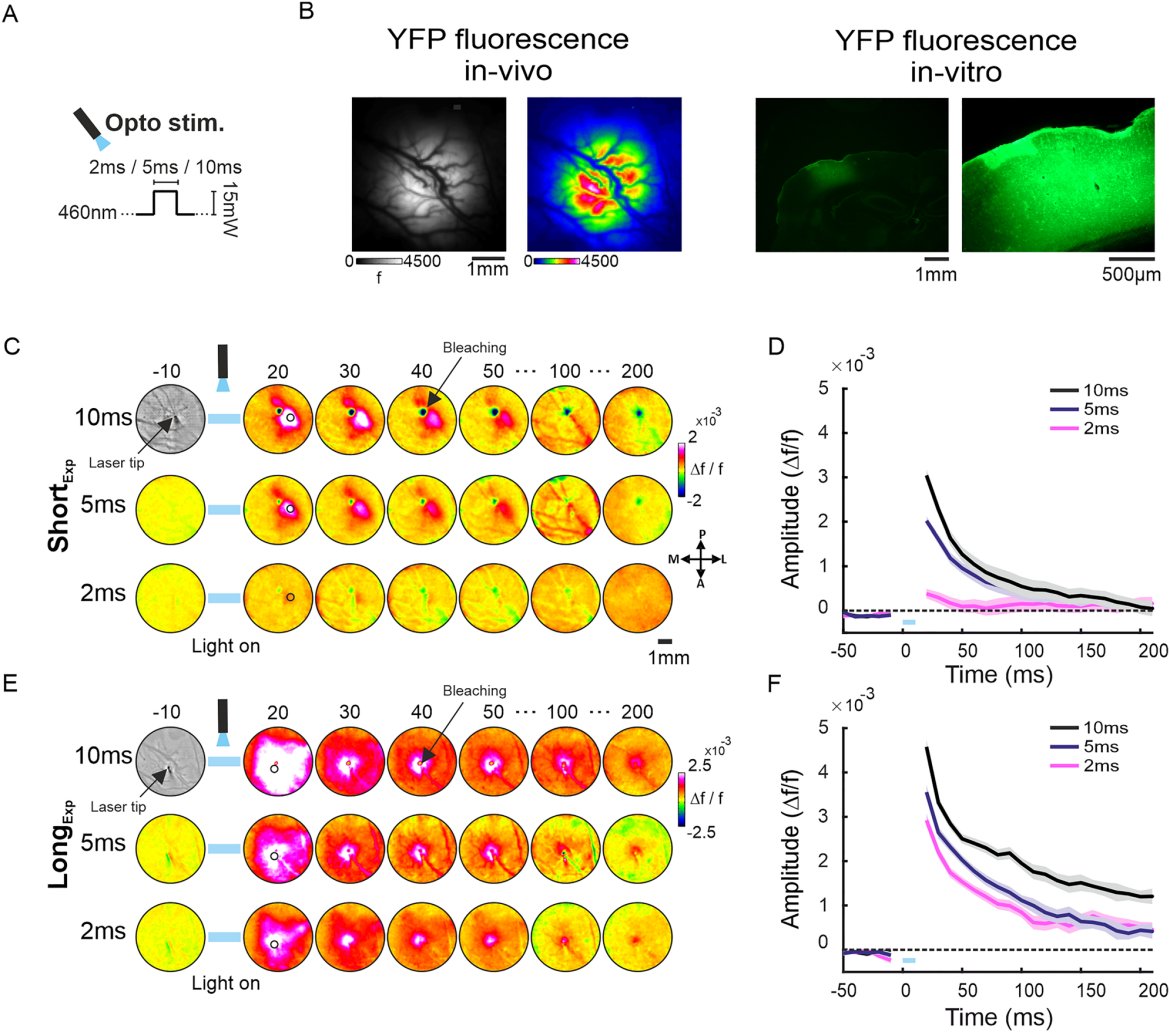
Example sessions of VSD maps evoked by different pulse duration of optostimulation. (**A**) Schematic illustration of the optostimulation parameters. (**B**) Left: in-vivo fluorescence images of ChR2-YFP expression in the imaged area, grey and color-coded maps (see "Methods"). Right: in-vitro fluorescence microscope images of ChR2-YFP expression (see "Methods") in cortical slices (performed verticaly to the surface of the imaged area). (**C**) VSD maps evoked by optostimulation in the barrel cortex expressing ChR2, imaged at 3–4 weeks after viral injection (Short_Exp_), maps are color coded. The numbers above the maps represent the time in ms relative to optostimulation onset. Blue horizontal bar indicates the optical artifact from the laser fiber. The circle ROI centered at peak VSD response is depicted on the 20 ms maps (black contour). A photo-bleaching effect is visualized as a darker spot pointed by an arrow (map = 40 ms; pixels with photo-bleaching were removed from further analysis). (**D**) Time course (TC) of the VSD signal averaged over the circle ROI pixels. Shaded area represents ± 1 SEM over trials (2 ms, n = 15; 5 ms, n = 19; 10 ms, n = 11). Blue bar above X-axis denotes the optostimulation duration. (**E**) Same as in C but for an optostimulation session performed at 8 weeks from injection (Long_Exp_). (**F**) same as in D but for Long_Exp_ condition (number of trials: 2 ms, n = 15; 5 ms, n = 14; 10 ms, n = 13).

For optogenetic experiments, the barrel cortex of 7 adult rats was injected with AAV5-CaMKIIa-hChR2(H134R)-EYFP (see "Methods"). The ChR2 is a light sensitive cation channel, that is expressed in the membrane of excitatory pyramidal neurons, due to the promotor type (CaMKII). Following 3–4 weeks (Short_Exp_) or 8 weeks (Long_Exp_) from viral injection we stimulated the barrel cortex with one pulse of a blue light laser (460 nm) at 15 mW. The optostimulation pulse duration was set to 2 ms, 5 ms or 10 ms, while we imaged the neuronal population response using VSDI (Fig. 1A and Supplementary Fig. S1A). The short optostimulation pulses were chosen to avoid possible side-effects emerging from heating of the cortical tissue by the laser (see "Methods";^29,30^). Because both optostimulation and VSDI techniques are based on optical signals, an optical artifact is visible during the period of laser stimulation (Supplementary Figs. S3–S4). Another phenomenon occurring due to the laser stimulation is photo-bleaching of the VSD, which is visible as a darker spot i.e., a lower signal around the tip of the laser fiber (for more details see Supplementary Figs. S3–S4). Therefore, we removed from data analysis the frames of stimulation (t = 0–10 ms) and pixels with photo-bleaching (see "Methods"). Finally, we performed an additional control experiment demonstrating that despite the laser artifacts, the neuronal response to whisker deflection can be detected reliably following optostimulation offset (for more details see Supplementary Fig. S5).

In 6 animals (out of 7), before staining the cortex with the VSD, we imaged the YFP expression *in-vivo* through the imaging window in addition to *in-vitro* verification of YFP expression (see "Methods"). Figure 1B shows ChR2-YFP expression in-vivo (left) and in-vitro (right). The *in-vivo* images are showing the YFP fluorescence over the imaged area while the fluorescence microscope images were taken post mortem to validate *in-vitro* the expression of ChR2-YFP. Brighter areas in the image indicate higher expression level of ChR2 (in animals with no YFP fluorescence or very low expression levels, no VSD evoked response signal was observed when stimulating the cortex with a laser pulse). To quantify the area of YFP expression we performed the following analysis on the YFP images taken in Short_Exp_ and Long_Exp_. First, the YFP image were normalized between 0 and 1, to account for the differences in the imaging conditions (see Supplementary material and Fig. S6). Next, we computed the number of pixels crossing a threshold of 2STDs from mean fluorescence value of the YFP image. Supplementary Fig. S6A shows two example maps, where the colored pixels denote pixels with YFP values crossing 2STDs above the mean YFP fluorescence image. The Long_exp_ map shows a higher pixel count than the Short_exp_. This is further quantified in Fig. S6B for all animals, and shows that the number of pixels was larger in the Long_Exp_ vs. Short_Exp_: Long_Exp_, 438 ± 52.9, Short_Exp_, 231.7 ± 92 (mean ± SEM). While the difference between the groups is not-significant (206 pixels; each pixel is 50^2^µm^2^), the value for shuffled data (mean over all random permutation) is 21 ± 131, which is 1.5 STD lower than the real data (Fig. S6B, inset). In summary, these results indicate that the YFP expression showed a trend toward larger values in the Long_Exp_.

Figure 1C, E shows the VSD maps evoked by short pulses of optostimulation for two example sessions: Short_Exp_ condition (Fig. 1C) and Long_Exp_ condition (Fig. 1E). In each session, we used 3 pulse durations: 2 ms, 5 ms and 10 ms. The fiber tip was placed above the cortical surface expressing the virus, according to the *in-vivo* fluorescence map of the YFP. The VSD maps in Fig. 1C show that immediately following the optostimulation offset (t = 20 ms) there is an increased VSD signal, corresponding to increased population response, spreading from the stimulation site horizontally over cortical surface (as explained above, closer to the fiber tip a photo-bleaching effect is visualized as a darker spot pointed by an arrow, map = 40 ms; see also Supplementary Figs. S3–S5). Shorter duration of optostimulation evoked lower population response amplitude that spread over a smaller cortical region, while this result occurred in both the Short_Exp_ (Fig. 1C) and Long_Exp_ (Fig. 1E) conditions. In the Short_Exp_, following optostimulation offset, the population activity quickly declined back to baseline while in the Long_Exp_ the activity declined more slowly, this difference was visible for all stimulation duration. Moreover, the VSD maps show that the evoked population response was higher for Long_Exp_ than Short_Exp_ condition. To further quantify this, we computed the time course (TC) of the VSD signal in a circular region of interest (ROI) centered at the peak cortical response (black circle contour superimposed on the maps = 20 ms in Fig. 1C, E; see Method). Figure 1D, F show the TC of the VSD signal for the different pulse durations and for the Short_Exp_ and Long_Exp_ conditions. The VSD TC shows a higher population response amplitude for the longer pulse duration relative to shorter pulse duration in each conditions (*p < 0.05, Wilcoxon rank-sum test), and general higher responses for the Long_Exp_ vs. the Short_Exp_ condition for the same stimulation duration (Short_Exp_: 2 ms, 0.38 ± 0.1 × 10^–3^, n = 15 trials; 5 ms, 2.02 ± 0.09 × 10^–3^, n = 19 trials; 10 ms, 3.04 ± 0.18 × 10^–3^, n = 11 trials; Long_Exp_: 2 ms, 2.9 ± 0.14 × 10^–3^, n = 15 trials; 5 ms, 3.5 ± 0.16 × 10^–3^, n = 14 trials; 10 ms, 4.6 ± 0.19 × 10^–3^; mean ± SEM; here and elsewhere; *p < 0.05, Wilcoxon rank-sum test).

**Figure 2:**
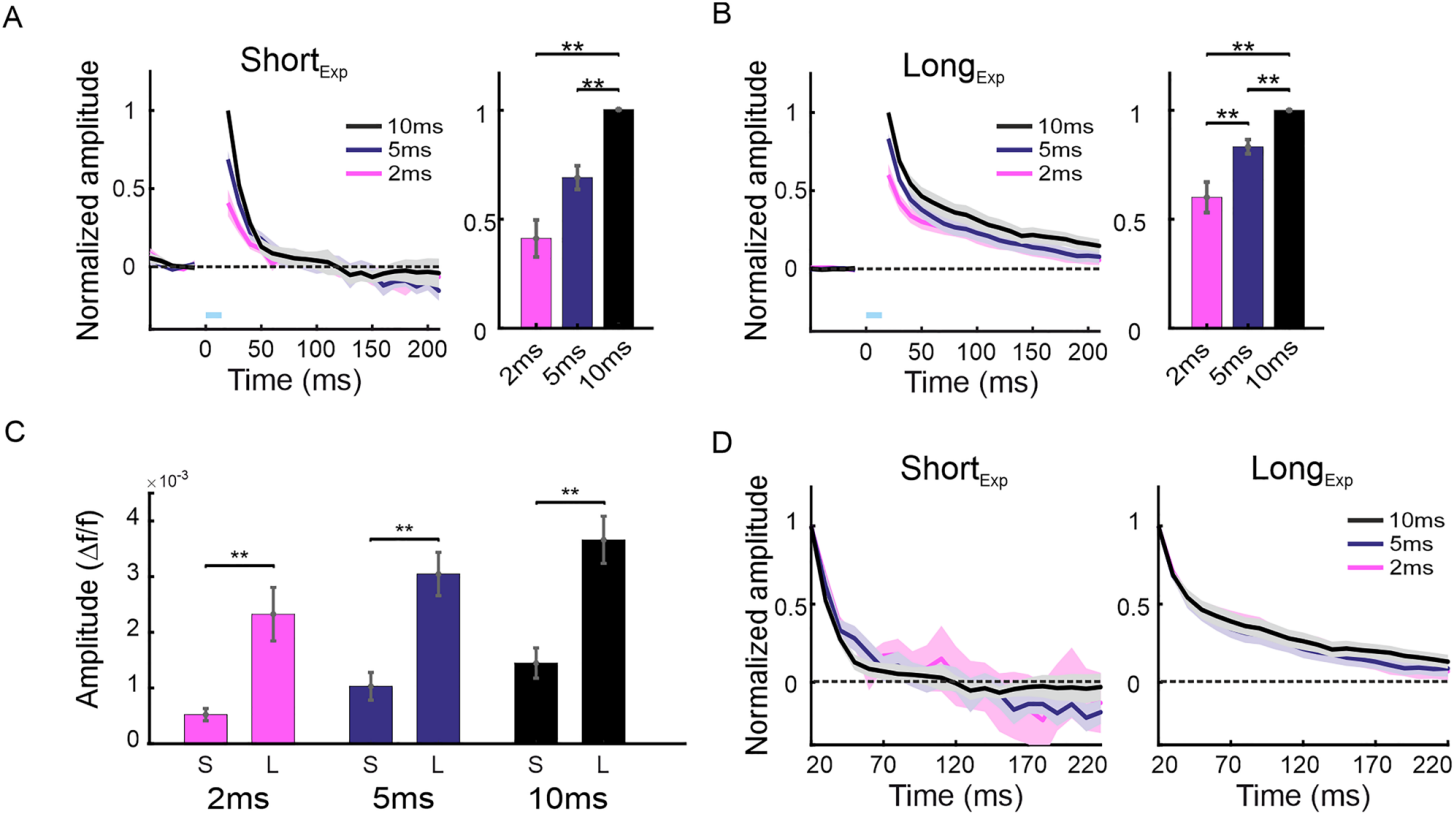
Grand average analysis for optostimulation: characteristics of population response for the different pulse durations and for short and long viral expression time. (**A**) Left: Mean normalized TC of the VSD signal over all Short_Exp_ sessions (2 ms, n = 6; 5 ms, n = 6; 10 ms, n = 8 sessions). In each session the VSD signal was normalized to the VSD response of 10 ms condition at the first frame after the laser artifact (20 ms post stimulus onset). The shaded area represents ± 1SEM over sessions. Blue bar above X-axis denotes the stimulation duration. Right: The mean normalized VSD peak response for the Short_Exp_ sessions. * *p* < 0.05, **p < 0.01, Wilcoxon rank-sum test. (**B**) same as in A but for Long_Exp_ sessions (2 ms, n = 6; 5 ms, n = 7; 10 ms, n = 7 sessions). (**C**) Comparison of the non-normalized VSD response at peak response, for the Short_Exp_ and Long_Exp_. Same data as in A and B, but non-normalized. (**D**) VSD TC normalized for each condition. Here, the VSD response of each condition, was normalized to its own peak response, this enables to compare the VSD TC dynamics across optostimulation duration, while accounting for the amplitude difference. The shaded area represents ± 1SEM over sessions. Left: Short_Exp_ sessions, Right: Long_Exp_ sessions.

Next, we computed the grand average population response (mean over all imaging sessions; Fig. 2). To account for the variance of the VSD response amplitude across sessions (emerging from: (i) variance across animals (ii) VSD staining quality etc.), in each session the VSD signal was normalized to the response amplitude of the 10 ms pulse (t = 20 ms; post stimulation onset). Then, population response was computed in in the ROI centered at peak response (as in Fig. 1) and averaged across all sessions (Fig. 2A, B). The grand average TCs in Fig. 2A, B show that the peak VSD response for longer optostimulation duration resulted in higher evoked response for Short_Exp_ and Long_Exp_ (Short_Exp_: 2 ms, 0.4 ± 0.08; 5 ms, 0.7 ± 0.05; 10ms, 1 ± 0; Long_Exp_: 2 ms, 0.6 ± 0.07; 5 ms, 0.8 ± 0.03; 10ms, 1 ± 0; **p < 0.01, Wilcoxon rank-sum test). Next, to compare the response amplitude differences between the two viral expression duration we computed the non-normalized mean peak response of each stimulation duration (Fig. 2C). For all stimulation durations the response for Long_Exp_ was significantly higher than Short_Exp_ (2 ms: 0.5 ± 0.1 × 10^–3^/2.3 ± 0.48 × 10^–3^; 5 ms: 1.03 ± 0.25 × 10^–3^/3.05 ± 0.4 × 10^–3^; 10 ms: 1.4 ± 0.27 × 10^–3^/3.6 ± 0.42 × 10^–3^; Short_Exp_/Long_Exp_ respectively; **p < 0.01, Wilcoxon rank-sum test).

Despite the VSD amplitude differences between pulse durations, when investigating the VSD decay dynamics of the different stimulation durations (by normalizing the VSD TC to the peak response of each stimulation duration separately), we found that the different pulse durations induced similar decline dynamics in both Short_Exp_ and Long_Exp_ (Short_Exp_, Fig. 2D left; Long_Exp_, Fig. 2D right). This result means that when accounting for the response amplitude difference, the dynamics of the VSD response is similar for the same stimulation duration, within each group (Short_Exp_ or Long_Exp_). Interestingly, Fig. 2D shows that the normalized response dynamics i.e. the decay rate is much slower for the Long_Exp_ expression. These results suggest a more efficient optostimulation activation after the longer expression period that may emerge from a higher expression of the opsin channels in the neuronal membrane.

**Figure 3:**
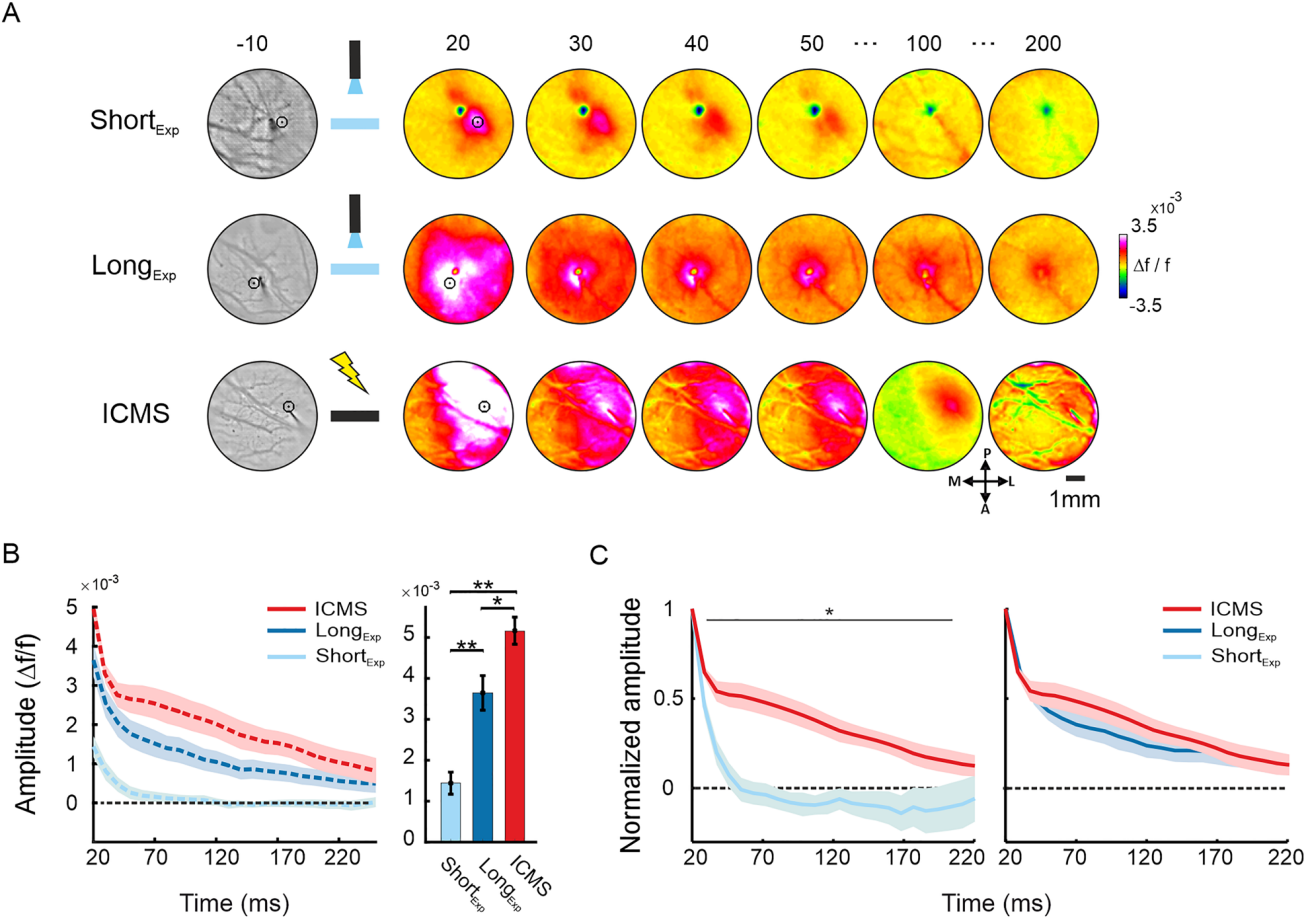
Comparison of the VSD response evoked by ICMS and optostimulation. (**A**) Population response maps evoked by optostimulation (10 ms) in the barrel cortex at 3–4 weeks after injection (ShortExp; top), 8 weeks after injection (LongExp; middle) or ICMS (no viral injection; bottom). The optostimulation sessions are the same as in Fig. 1A (note that the map clipping is different for comparison purposes with ICMS). (**B**) Left: TC of the VSD response to ICMS (red), ShortExp (light blue) and LongExp (blue) optostimulation sessions average over a small circle ROI centered at peak response. The shaded area represents ± 1SEM over sessions (ICMS, n = 6; ShortExp, n = 8; LongExp, n = 7). Right: response amplitude at 20 ms post stimulus onset for the TC depicted in the left panel. **p < 0.01, Wilcoxon rank-sum test. (**C**) Same TCs as in B, but normalized to the VSD amplitude at 20 ms of each condition. This enables to compare the decay rate (i.e. the dynamics) of the VSD response over conditions while accounting for differences in peak amplitude. Left: comparison of the VSD signal evoked by ICMS and ShortExp. Right: same as in left, but for ICMS and LongExp.

Next, we wanted to investigate whether the optostimulation activates the neuronal cortical network in a similar manner to that of the ICMS. For this comparison we used the 10 ms pulse duration of the optostimulation experiments, because they provide better SNR responses than shorter pulse durations. For electrical microstimulation in the barrel cortex of naïve rats we used a short microstimulation pulse (20 ms; see "Methods"; Fig. 3A bottom and Supplementary Fig. S1B) that evoked a VSD response with a temporal profile comparable to that of the optostimulation (i.e. a short rising population response peaking at 20 ms post stimulation onset; for more details see^22^, Fig. 2). The ROI in both experiments was selected in a similar manner: a circular ROI centered at the peak response (Fig. 3A). Although for ICMS we can image neuronal response from stimulation onset (t = 0), because no optical artifacts are generated when using ICMS, for the comparison purposes, we performed all the analyses from the time of optostimulation artifact offset (t = 20 ms), which is also the time of ICMS offset.

Figure 3A shows example sessions for VSD response maps evoked by optostimulation in Short_Exp_ (top; same as in Fig. 1C) or Long_Exp_ (middle; same as in Fig. 1E) and ICMS (bottom). The VSD maps show that ICMS evoked a widespread increased population response, spreading horizontally over the cortical area from the electrode tip and decaying slowly back to baseline. This widespread response and the slower dynamics are more similar to the population response maps of the optostimulation in the Long_Exp_ (compare Fig. 3A middle to bottom). To further investigate this, we first computed the TC of the VSD signal in the peak response ROIs, for all sessions in the ICMS (red curve), Long_Exp_ optostimulation (blue curve) and Short_Exp_ optostimulation (light blue curve; Fig. 3B left) conditions. The population response in the ICMS shows a slightly higher peak response near the electrode tip relative to Long_Exp_ optostimulation, while both show significantly higher peak response than the Short_Exp_ condition (Short_Exp_: 1.4 ± 0.27 × 10^–3^; Long_Exp_: 3.6 ± 0.4 × 10^–3^; ICMS: 5.1 ± 0.3 × 10^–3^; *p < 0.05, ** p < 0.01; Wilcoxon rank-sum test; Fig. 3B right). To compare the decay rate (i.e. TC dynamics) of the VSD response and account for variance of the VSD response amplitude across experiments, the VSD signal was normalized to the response at t = 20 ms (post optostimulation or ICMS onset). Figure 3C shows the TC of the normalized responses at the peak response ROI for ICMS relative to Short_Exp_ (left) or Long_Exp_ (right). The decay rate of the VSD response to ICMS near electrode tip is more similar to the VSD response evoked by Long_Exp_ optostimulation and it is much slower than Short_Exp_ (*p < 0.05; Wilcoxon rank-sum test).

**Figure 4:**
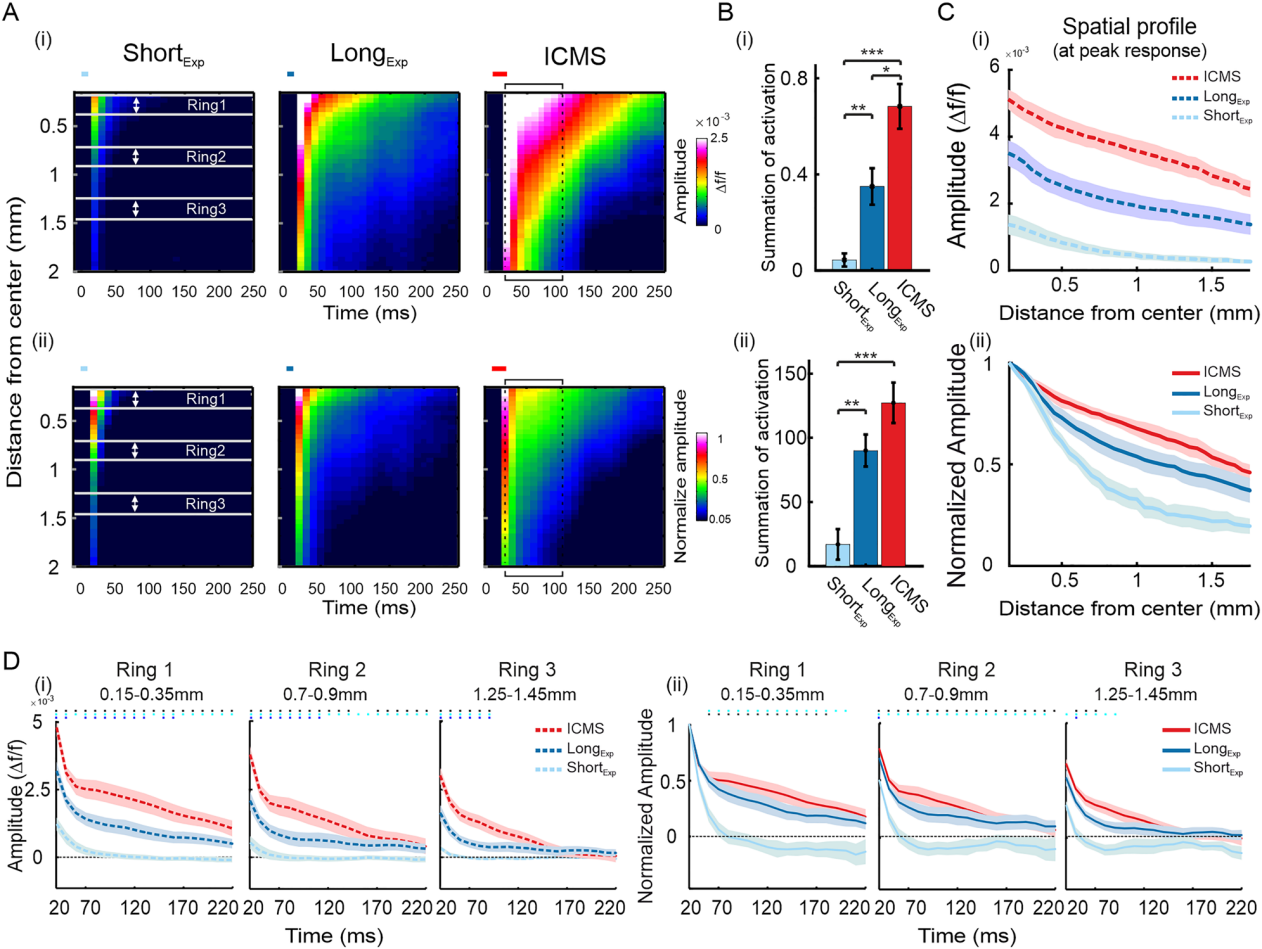
Comparison of space–time analysis in ICMS and optostimulation. (i) Denotes analysis for non-normalized data. (ii) denotes analysis for normalized data. The VSD response was normalized to peak response at the center (defined as a circle ROI with r = 150 µm) of the ring analysis (see "Methods" and supplementary Fig. S7), for optostimulation or ICMS conditions. (**A**) Space vs. time plots: the population response at increasing distances from the peak activation (center ring), as function of time. (**B**) Sum of activation over space and time for the maps depicted in B. The summation window is illustrated as a black contour on the ICMS maps in A. *p < 0.05, **p < 0.01, Wilcoxon rank-sum test. (**C**) Mean spatial profile of the VSD responses, over all sessions, at time of peak response. (**D**) TC of the VSD response at increasing distance from the center (i.e. peak response). The rings location over space are depicted on the left maps in A as white lines (ring1: 0.15–0.35 mm from peak response; ring2: 0.7–0.9 mm from peak response; ring3: 1.25–1.45 mm from peak response). The asterisks above TCs represent a significant deviation of ICMS from Short_Exp_ condition (red) or from the Long_Exp_ condition (black). The deviation of Long_Exp_ from Short_Exp_ optostimulation is marked with blue asterisks. Wilcoxon rank-sum test: *p* < 0.05.

Next, we wanted to compare the spatial activation pattern of the evoked population response in the ICMS and optostimulation experiments. To investigate the spatial spread of cortical response evoked by Short_Exp_ or Long_Exp_ optostimulation and to compare it to ICMS we performed a space–time analysis. We generated a continuous set of non-overlapping rings, centered over the peak VSD response. The rings’ size increased from the center to 2mm radius at steps of 50 µm (one pixel) for each ring (see Supplementary Fig. S7). Next, we computed the VSD response amplitude for each ring (mean over ring's pixels) on each frame and obtained space–time maps. Figure 4Ai shows the space–time maps average over sessions: Short_Exp_, left; Long_Exp_, middle; ICMS, right. The maps show the population response amplitude as function of distance from peak response in space which is also the center of all rings (y-axis; distance from center) for each time point (x-axis) starting at 0.15mm from the center. While the Short_Exp_ condition shows a lower population response and limited spatial spread, the ICMS shows a much larger response and spread of activation over space and time. The Long_Exp_ condition shows an intermediate state: a substantial spatial spread with higher response amplitude relative to the Short_Exp_ but still lower than the ICMS condition. To quantify this, we computed the sum of overall population activation in each session for every stimulation type separately over space (up to 2 mm from the center) and time (from t = 20 ms up to 100 ms from stimulation onset; see the black square contour on ICMS map, Fig. 4A right). The summed activation (Fig. 4Bi) of ICMS is higher than Long_Exp_ (Long_Exp_, 0.35 ± 0.08; ICMS, 0.68 ± 0.09; *p < 0.05) while both are significantly higher than Short_Exp_ expression (Short_Exp_, 0.04 ± 0.03; **p < 0.01, ***p < 0.001; Wilcoxon rank-sum test). The spatial profiles in Fig. 4Ci show that the amplitude differences between stimulation types are maintained across cortical distance (from the central ring to the most outer ring, 2mm further). The larger spatial spread of population activation in the Long_exp_ (compared with the Short_exp_) is in accordance with the larger area of YFP expression in the Long_exp_ condition (Fig. S6A, B; in comparison with the Short_EXP_ condition). The higher peak VSD response in Long_exp_ vs. Short_exp_ is in accordance with the higher YFP expression in the same ROIs (Fig. S6C).

Next, to investigate the response dynamics over time and space, rather than amplitude differences, we normalized the response in each ring to the peak response at t = 20 ms of the center ring (Fig. 4Aii). This normalization enables to compare the spatio-temporal profiles across the different conditions, after accounting for the peak amplitude differences over the conditions. While the normalized space–time maps of the Short_Exp_ shows only a small spread of activity over space, the Long_Exp_ shows an intermediate state that is more similar to the ICMS maps (summed activation: Short_Exp_, 18.36 ± 12.03; Long_Exp_, 91.44 ± 12.99; ICMS, 131.6 ± 16.42; **p < 0.01 and ***p < 0.001; Wilcoxon rank-sum test; Fig. 4Bii). Moreover, the spatial profiles of the ICMS and Long_Exp_ were comparable (red and blue curves in Fig. 4Cii), mostly at short distance from the center ring i.e. peak VSD response. Both conditions showed a slower decay over cortical distance relative to Short_Exp_ (both showing significantly higher response amplitudes at larger distances from the center than Short_Exp_; *p < 0.05; Wilcoxon rank-sum test; Fig. 4Cii). Together, these results suggest variation in the spatial spread over cortical space for the different simulation types, while the Long_Exp_ optostimulation showed an intermediate state with higher similarity of the activation pattern to ICMS. To further investigate this, we studied the TC of the VSD signal at three different distances from the peak response (Fig. 4D; Rings are illustrated by white lines in Fig. 4A left map). The VSD signal in the Short_Exp_ optostimulation shows lower amplitudes (Fig. 4Di, light blue curves) and faster response decay to baseline (Fig. 4Dii) than ICMS and Long_Exp_ at all three rings (small light blue asterisks above the graph denote significant difference of ICMS from Short_Exp_; small blue asterisks denote significant difference of ICMS from Long_Exp_; small black asterisks denoted significant difference of Long_Exp_ from Short_Exp_ with *p* < 0.05; Wilcoxon rank-sum test). ICMS (red curves) shows higher response activation than Long_Exp_ (dark blue curves) at all three rings (Fig. 4Di), yet similar rate of response decay in rings 1–2 (Fig. 4Dii). At more remote cortical areas, i.e. ring 3 (Fig. 4Dii) ICMS shows a slower rate of response decay relative to the Long_Exp._ In summary, the spatio-temporal patterns of the VSD response in the Long_Exp_ optostimulation showed an intermediate state between Short_Exp_ and ICMS conditions with more comparable spatial profiles and dynamic to the ICMS condition, mainly around the center.

**Figure 5:**
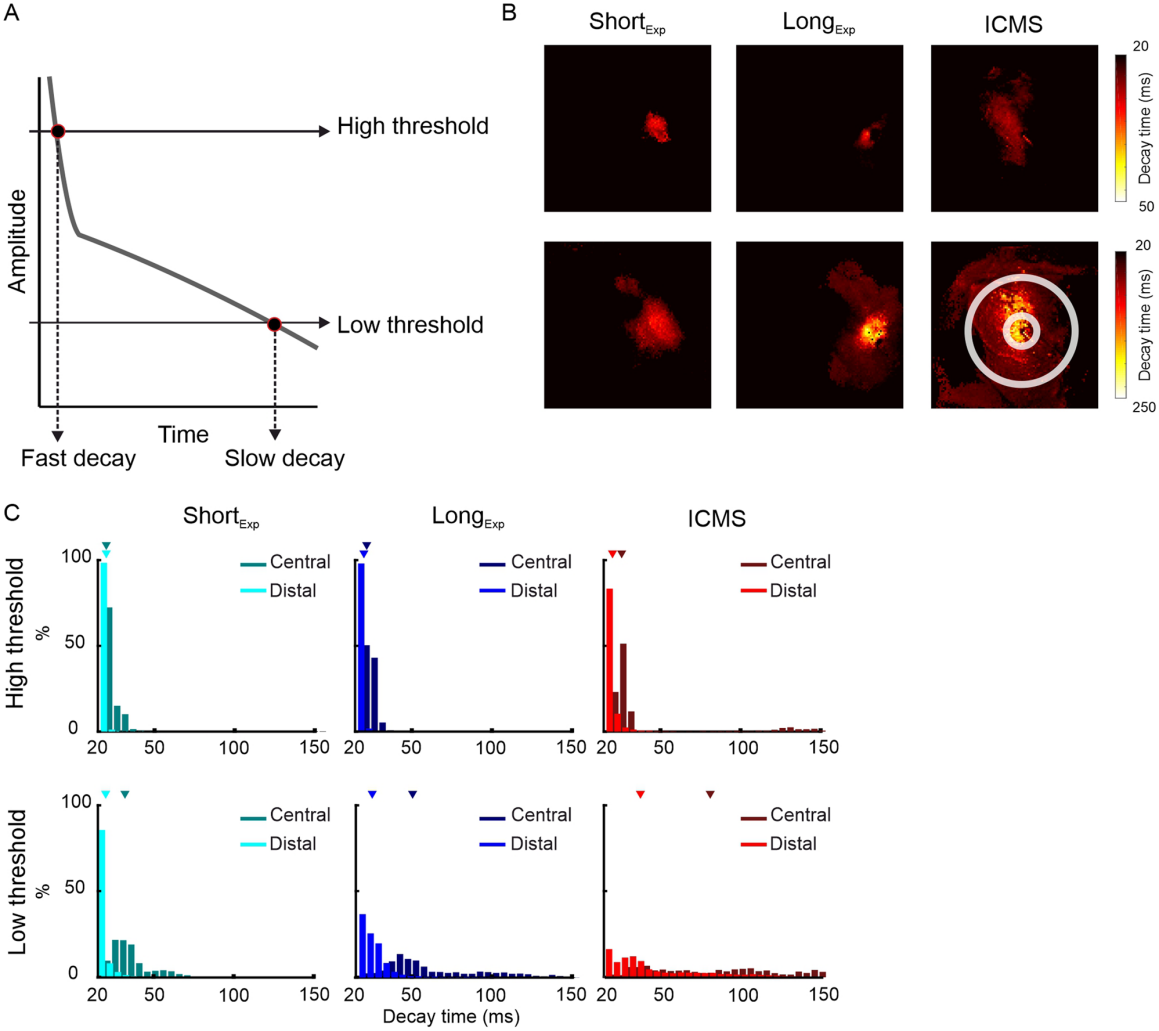
Maps of decay time to threshold. (**A**) Illustration of a typical TC of the VSD response to ICMS. The upper horizontal line represents a high threshold and the lower horizontal line represents a low threshold. (**B**). Example time to threshold decay maps (see "Methods") for Short_Exp_ (left), Long_Exp_ (middle) and ICMS (right). Top row denotes decay maps to high threshold (70% from peak response) and bottom row denotes decay maps to low threshold (30% from peak response). The values of decay time are color coded. White circle contours over right bottom map illustrate pixels close or far from the stimulation site by a microelectrode or laser fiber tip (central and distal; corresponding to ring 1 and ring 3 in Fig. 4, respectively). (**C**) Distribution histograms of the decay time in central and distal pixels for all sessions. The triangles show the median decay value. Top rows show the distributions for high thresholds and bottom row shows the distributions for low thresholds.

The decay of the VSD signal back to baseline shows two phases: an early fast decay followed by a slower decline (Figs. 4Di, 5A). To quantify this over cortical space, we computed for each session a map of the decay time to a threshold amplitude (see "Methods"), where each pixel depicts the decay time to cross a threshold from peak VSD response. To characterize the two decay rates, we computed two sets of maps: a high threshold map (threshold = 70% of peak response) and a low threshold map (threshold = 30% of peak response). Figure 5B shows example maps for each stimulation type and expression duration. For the high threshold (Fig. 5B top), all stimulation conditions showed a fast decay rate, i.e. short latencies, for pixels at the vicinity of the stimulation site. For the low threshold (Fig. 5B, bottom), the ICMS and Long_Exp_ maps showed a wider latency distribution: longer latencies characterize pixels near the stimulation site and shorter latencies characterized more remote pixels. In contrast, the Short_Exp_ showed mainly fast decay. To further quantify the decay rate of the VSD signal at the center and remote regions we defined two rings ROIs, a central and a distal ROI relative to stimulation site (same as ring 1 and ring 3 as in Fig. 4). Figure 5C shows the distribution of decay time to threshold, for all central (dark colors) and distal (bright colors) pixels from all sessions. For the high threshold distributions, all stimulation conditions showed a fast decay rate for the central and distal pixels (Fig. 5C top; median decay time denoted by triangles: Short_Exp_, central/distal 20 ± 3.2/20 ± 0.26; Long_Exp_ center/distal, 22.61 ± 2.5/20 ± 0.27; ICMS, central/distal 25.31 ± 2.9/20 ± 2 ms; median ± mad). For the low threshold, the ICMS and Long_Exp_ show a VSD signal with much slower decay rate with wider distribution at the center than Short_Exp,_ that was characterized by narrower distributions with shorter decay values at central and distal pixels (Fig. 5C bottom, median decay time denoted by triangles: Short_Exp_, central/distal 32.02 ± 9.8/20 ± 2.6; Long_Exp_ center/distal, 51.27 ± 25.7/26.1 ± 6.5; ICMS, central/distal 82.43 ± 32.6/36.37 ± 20.17 ms; median ± mad). In summary, the decay time histogram in the Long_Exp_ condition showed an intermediate state between the Short_Exp_ condition and the ICMS condition, with higher similarity to microstimulation near stimulation site.

## Discussion

In this work we investigated the spatio-temporal patterns of population response evoked by a single pulse of optostimulation and compared it to ICMS. For optogenetic experiments, we used a viral injection to express ChR2 in excitatory neurons of the barrel cortex and then stimulated the cortex with a blue laser, at 3–4 weeks (Short_Exp_) or 8 weeks (Long_Exp_) following the transfection. We found that longer optostimulation duration resulted in higher population response, for both expression periods. However, optostimulation in the Long_Exp_ condition induced higher and more prolonged evoked response than in the Short_Exp_ condition. Next, using a microelectrode, inserted into the upper layers of the barrel cortex, we applied ICMS in a different group of rats. The VSD response in the Long_Exp_ condition showed an intermediate state that was more similar to the ICMS activation patterns, although the population response at remote sites relative to the stimulation site showed a faster decay rate.

Only very few studies investigated the VSD response evoked by ChR2 stimulation, at both the subthreshold and suprathreshold level of population activity, over space and time^31–33^. In this study, we aimed at measuring the population response in the barrel cortex of adult rats expressing ChR2, after using viral transfection to excitatory neurons in the upper cortical layers. The combination of 460 nm laser stimulation with VSDI in-vivo resulted in several limitations. First, as has been reported before^34^, light from the laser stimulation penetrated the optical filters causing light artifacts (despite the existence of an emission filter and dichroic mirror, placed in the light path between the cortex and the camera). While the laser stimulation was ON, a substantial optical artifact was detected by the camera’s sensors (see Supplementary Fig. S2). To overcome this problem, we analyzed the VSD data after the light stimulation was turned off. Thus, the combination of VSDI with optical stimulation holds a similar limitation to combining electrical stimulation with electrophysiological recordings. Another way to minimize this drawback, is to combine optostimulation with electrophysiological recordings. This approach will enable to fill part of the missing information, mainly during the rising phase of the response and during the ICMS itself, but it will not enable to obtain it at high spatial resolution. Second, the combination of VSD and laser stimulation resulted in photo-bleaching of the blue dye at the site of stimulation (see Supplementary Figs. S3–S5). Lim and his colleagues stimulated ChR2 in transgenic mice and imaged the neural responses with VSDI. In accordance with our results, they observed VSD bleaching at the site of optical stimulation and further attempted to avoid this artifact in their analysis^32^. We found that the bleaching artifact over the cortex increased with stronger or longer optostimulation (see supplementary Figs. S3–S5). Thus, to measure the neural response as close as possible to the stimulation site, we used short stimulation duration which induced a smaller bleaching artifact. Moreover, we used a short optostimulation at low power to minimize possible heating artifact of the cortical tissue (these parameters were reported to induce minor temperature changes of up to 0.01 deg^30^.

We delivered a brief pulse (up to 10ms) of laser stimulation to the barrel cortex following two expression periods: Long_Exp_ and Short_Exp_. First, we found that optostimulation in the Long_Exp_ condition resulted at higher VSD amplitudes relative to Short_Exp_ condition (Figs. 1, 2). Second, we found that while Short_Exp_ condition showed a fast decay rate and small spread of cortical evoked response, Long_Exp_ condition results with prolonged evoked responses and spread over a wider region (Figs. 2, 4). A Previous study showed that the VSD signal of 1ms optostimulation delivered to the sensory cortex of mice showed a fast response decay (within 25ms after stimulus onset^32^). Similar fast decline of the evoked response was observed in an electrophysiological study (following 30 ms stimulation)^35^. What is the source for the different spatio-temporal pattern of neuronal activation in the Short_Exp_ and Long_Exp_ coditions? To deliver ChR2 to the cortical tissue we used the recombinant adeno-associated viral vector type 5 (AAV5), which over time, generates increasing expression levels in cells at the injection site^36^ during the first 2 months after infection^37,38^. Zhang et al.^39^ reported that to reach a high steady-state levels of ChR2 expression at distal axonal processes, longer periods of expression (> 6 weeks) are necessary^39^. Therefore, it is reasonable to assume that in the Long_Exp_, in comparison to Short_Exp_ condition, the expression of ChR2 in the cell membrane and also in the axonal processes (of the transfected cells) will be higher. Thus, an optostimulation pulse in the Long_Exp_ condition will excite a larger area of neuronal membrane, including more cells and axons, which will result in higher and larger spatial spread of population activation.

In the current study, we stimulated the barrel cortex using microstimulation or optostimulation and compared the evoked responses. We compared the population response evoked by a 10 ms laser pulse in optostimulation to microstimulation of 20 ms. As the closing tau of the ChR2(H134R) is ~ 17 ms, the effective opening duration of the channels is longer than the optostimulation pulse itself (10ms), approaching 20ms, which is more similar to the ICMS train duration (Figs. 3, 4). ICMS and optostimulation were suggested to stimulate the neural network in a different manner by activating different neural elements. ICMS activates the neural network in un-specific manner, excitatory and inhibitory neurons and mainly affect axons of passage near the microelectrode, leading to a large spread of response and activation of neurons as far as few millimeters away from the stimulation site^12,23,40–42^. Optostimulation, on the other end, engages mainly cell bodies and based on the promotor can activate specific population of cells. The CaMKIIα promotor used in this study, enables to stimulate directly only excitatory pyramidal neurons. However, because the pyramidal neurons project to inhibitory neurons, they can recruit also the inhibitory network, as occurs in microstimulation^23^. Recently, the usage of pan-neuronal promoters in AAV5 was successfully applied in the somatosensory cortex of rats^19^ and this approach may enable to further compare the spatio-temporal patterns of population responses evoked by ICMS and optostimulation.

We next asked: what can cause the similarity of population response evoked by ICMS to Long_Exp_ in comparison to Short_Exp_? First, longer expression periods will result in higher expression of ChR2 at the cell membrane, as well as at the axonal membrane. Second, AAV5 can undergo retrograde transport from nerve terminals at the site of injection which can lead to the transfection of neurons whose cell bodies are remote from the injection site^43^. Therefore, optostimulation at the expression site after 8 weeks from the injection, will activate not only more cell bodies at that injection site and their axons, but also axons and cells bodies that are located remotely from the injection site, which is more similar to how microstimulation activates the network. Figure 5 showed that the VSD signal has two decay components: while the slow decay appeared only in ICMS and Long_Exp_ conditions, fast decay characterized all stimulation conditions. What are the neuronal mechanisms that underlie the slow and fast decay? The fast decay may result from local cell bodies excitation and the slow decay may result from multi-synaptic activity propagation through short horizontal connections. This is in accordance with Fehérvári et al.^44^ that reported also on slow and fast decay in the VSD signal following ICMS in the visual cortex of mice. In this study, the fast decay characterized the low intensity ICMS and the slower decay appeared at the higher intensity ICMS. Their interpretation was similar to our: higher current stimulated a large number of neurons around the electrode tip simultaneously and synchronized their spiking, thus induce an effective regenerative wave across multiple synapses through short horizontal connections, while the weaker stimulus was less likely to activate a sufficient number of neurons to induce a regenerative travelling wave. In addition, Kumaravelu et al.^45^ investigated the spatial effects of ICMS in a brain model simulating a single cortical column. Interestingly, and in accordance with our interpretation, they found that ICMS at high current amplitude, induce mainly axonal activation. Finally, we note that Millard et al.^24^ compared the evoked responses in the barrel cortex following electrical stimulation and optostimulation in the thalamus, where the viral expression period was short (3–4 weeks). They reported a more localized response following optostimulation relative to ICMS. Interestingly, similar to that study, we showed that for the ICMS condition, the VSD evoked response lasted longer in time and propagated to more remote region in the cortex as compared with Short_Exp_ optostimulation. However, for longer viral expression period (Long_Exp_) the evoked response is larger and more similar to ICMS.

An important translational goal of neuroscience is to develop a brain-machine interface (BMI) that will artificially activate sensory and motor brain areas that can serve as neural substitution in human patients. Electrical stimulation is the main stimulation technique used in this field^46–48^. While ICMS evoke electrical activity in an unspecific manner and over large neuronal population, optostimulation can induce population specific and potentially more focal activation of neuronal population. However, our results reveal a more complex picture: we found that the optostimulation pattern of activation depends on viral expression duration. At longer periods of viral expression, optostimulation can activate not only cell bodies but also crossing fibers which can lead to network activity propagation through short horizontal connections, a neural mechanism that was reported for ICMS. This may be an important factor when planning a neuro-prostheses, because the expression level of the channel is expected to increase with time. Moreover, a useful neural-prostheses needs to activate the neural network in a similar manner to that evoked by physiological sensory stimulation. In a previous study, we compared a short whisker deflection to ICMS in the barrel cortex (see Fig. 8 at^22^) and we found that sensory stimulation shows similar population TC as the ICMS condition. The TC of population response in both stimulations conditions (whisker deflection and ICMS) was comprised from fast and slow decay phase, which seems to reflect a multi-synaptic propagation through short (and possibly also long) horizontal connections. This raises the possibility that optostimulation in the Long_Exp_ condition, in our experiments, evokes neural activity that is more similar to physiological sensory stimulation.

## Methods

### Experimental animals

13 male albino rats (Sprague Dawley, 200–500 g) were used for the experiments. For optogenetic stimulation 7 rats were used and 6 rats for ICMS. The ICMS data was used in a previous paper^22^, and here it was re-analyzed differently for a comparison with optostimulation. All experimental and surgical procedures were carried out according to the NIH guidelines, approved by the Animal Care and Use Guidelines Committee of Bar-Ilan University and supervised by the Israeli authorities for animal experiments. All the animal study information were carried in accordance with the recommendations in ARRIVE guideline^49^.

### Surgical procedure

#### Viral injection

For viral injection procedure, rats were anesthetized with Ketamin/Xylazin (100/20 mg/kg) and then placed in a stereotaxic apparatus. After skin incision, we performed a very small craniotomy through the skull over the barrel cortex in the left hemisphere (2mm posterior and 6mm lateral from the bregma) and removed the dura matter. Next, we performed a microinjection of the viral agent at 1–2 depths, (0.5 mm to 1 mm from the cortex surface) at perfusion speed of 80nl/min. At one depth experiments we injected 1.5 µl of virus while for the two depth experiments, we injected 1µl of virus to each depth. During the surgical procedures the rat's body temperature was maintained using a heating blanket and its eyes are protected with eye ointment. At the end of the surgery, the rats were treated with Ceftriaxone (20 mg/kg) and Dexamethasone (2mg/kg) for 3 days to prevent infection and reduce inflammation. In rodents brain, the opsin gene expression reaches functional levels in ~ 3 weeks after AAV injection^39^. The barrel cortex was infected with Adeno-Associated virus serotype 5: AAV5-CaMKIIa-hChR2 (H134R)-EYFP. The ChR2 (H134R) is maximally activated at 470 nm and has fast dynamics for channel opening (tau =  ~ 1.92ms) and slower dynamics for closing (tau =  ~ 17.9 ms)^50^. This viral vector construct has already been used successfully to transfect cortical neurons in rats and express ChR2^51^.

#### Chamber for optical imaging

For imaging experiments, rats were anesthetized with Urethane (1.5 g/kg), which provides a long-lasting stable anesthesia. After skin incision, a chamber was cemented to the skull and a craniotomy was performed over the left barrel cortex (stereotactic coordinates: 2mm posterior and 6 mm lateral of the bregma). Next, the dura mater was removed in to expose a ~ 5mm × 10 mm imaging area over the barrel cortex (for more details see^22^).

### VSDI Imaging

VSDI was performed in the rats under Urethane anesthesia (for more details see^22^). In brief, the cortex was stained with a VSD solution (0.75mg/ml, Oxonol VSD RH-1883) for 2h, then the cortical surface was washed with ACSF until the drain solution is clear. To enable imaging, we fill the chamber with transparent agar. VSDI was performed using the Micam Ultima (Scimedia, Japan) system based on a sensitive fast camera, which offers a resolution of 100 X 100 pixels and up to 10 kHz sampling rate. The exposed cortex is illuminated using an epi-illumination stage with an appropriate excitation filter (peak transmission 630 nm, bp10) and a dichroic mirror (DRLP 650), both from Omega Optical, Brattleboro, VT. To collect the fluorescence and reject stray excitation light, we place a barrier post-filter above the diachronic mirror (RG 665, Schott, Mainz, Germany). To obtain the vascular pattern of the cortex, we take an image of the cortex while illuminating it with a green light (540 nm bp10). Next, VSDI data acquisition can be performed for the next 3–4 h. We collect images of 100 × 100 pixels from a brain area of 5 × 5mm, each pixel thus corresponds to a cortical square area of 50^2^µm^2^.

### Stimulations

#### Whisker stimulation

To validate that we can image from the barrel cortex we stimulate the rats' whisker with a Piazo device in 10Hz (50ms stimulation followed by 50 ms interval). The Piazo is connected to the whisker of interest and is placed ~ 5 mm from the mystacial pad. In each session only one whisker is deflected along the horizontal axis.

#### Optostimulation

A 460 nm laser pulse (Omicron laserage, LightHUB-4; 200 µm diameter optical fiber) was used to stimulate ChR2-expressing neurons in the rats’ barrel cortex. We stimulate the brain with one square pulse of 2 ms, 5 ms, or 10 ms duration and laser power level was set to 15 mW. These parameters were used because: (i) using these parameters in naïve animals (i.e. no viral injection), we did not observe the development of a hemodynamic response (when using optical imaging of intrinsic signal, OIIS) around the laser fiber tip. The existence of a hemodynamic response may imply on neural activation or overheating of the brain tissue due to laser stimulation. In contrast, at higher intensities of laser power, or much longer duration, OIIS showed a development of a hemodynamic response (data not shown) (ii) Previous studies reported on the safe optostimulation parameters (duration and power) that did not produce heating effects^30^. Before starting VSDI experiment in the injected animals, we measured in-vivo the YFP expression in the barrel cortex (in 6 out of 7 rats) by imaging the florescence signal (480 nm ± 10 nm excitation filter and 530 nm emission filter; Fig. 1B). The laser’s fiber tip was positioned above the YFP expression location (see more information in Supplementary Fig. S1A), at minimal distance from the cortical surface, but it was not inserted into the cortical tissue. In one animal, YFP expression map was not available, thus fiber tip was positioned above the injection site. The laser stimulation most probably activated mainly neuronal elements (cells and fibers) in layer 2/3 (located at 150–550 µm from cortical surface. Narayanan et al.^52^ showed that most of light intensity from the laser fiber tip arrives to 100–150 µm below the tip and only 20% of light intensity arrives to 550 µm^53^.

#### Intracortical micro-stimulation (ICMS)

The microelectrode was targeted to the location barrel field (as detected using whisker stimulation) and inserted into the upper layer of the barrel cortex (~ 300µm). Biphasic square pulses were delivered to the barrel cortex through a standard tungsten microelectrode (FHC, Bowdoin, ME, USA; with an impedance of 300-600KΩ, tip end diameter is ~ 2 µm). Each biphasic pulse is composed from a cathodal (0.2 ms) pulse followed by an anodal (0.2 ms) pulse. We stimulated the brain with current amplitude of 80µA in 10 pulses at 500Hz (20ms stimulation duration; Fig. S1B). The output current from the microstimulation box was verified as a voltage measurement across a 100 KΩ resistor (see more information in supplementary Fig. S1B).

While we used tungsten microelectrodes for microstimulation, it is very unlikely that the microelectrode became less effective due to high charge flow in our experiments. The reasoning for this is as following: (i) The microstimulation used in our VSDI experiment (train of 20 ms, 500 Hz, 80 µA) was applied only once in a single trial, while single trials were separated by a 10 s interval (due to the VSDI protocol^54^). This leads effectively, to very short microstimulation duration with very long inter-trial intervals. (ii) In each VSDI experiment the microelectrode tip was examined under the microscope, at the beginning and end of each VSDI experiment. We did not observe changes in the shape of the microelectrode following the microstimulation. (iii) Throughout the experiment, the output current from the microstimulation box was continuously monitored as a voltage measurement across a 100 KΩ resistor located between the animal and the microstimulation box. (iv) Finally, if the microelectrode deteriorated during the experiment due to heavy charge flow, a reasonable prediction is that the evoked neural response will decrease over trials (as a result of the expected decrease in charge flow). We tested this hypothesis by comparing the VSD TC of the first and last trials across all ICMS sessions. We found that the VSD TC of the first and last trials were essentially identical and there is no significant difference in peak response or amplitude of the slow decay phase (*p* > 0.05).

### Histology

In addition to measuring the YFP expression in-vivo, we wanted to validate cortical YFP expression in-vitro, using histological methods. Thus, immediately after the last imaging session, rats were perfused intracardially with isotonic buffered saline followed by 4% paraformaldehyde in phosphate-buffer saline solution. The brain was removed to 30% sucrose solution in paraformaldehyde for several days. Next, the brain was frozen and subsequently sectioned into 50 µm coronal slices using a cryostat apparatus. Fluorescence images were taken with a fluorescence microscope (Nikon eclipse E400) to validate in-vitro opsin expression in the transfected area (excitation: 465–495 nm; Dichroic-mirror: 505 nm; emission: 515–555 nm).

### VSDI analysis

All data analyses were done using MATLAB software. The basic analysis of the VSD signal is detailed elsewhere (Ayzenshtat et al.^26^, see there supplementary Fig. 12; Margalit and Slovin^22^). Briefly, in each trial, the fluorescence level of each pixel is divided by background fluorescence of that pixel, which is the average fluorescence before stimulation onset^55^; also known as frame-zero division). To remove slow fluctuations in the VSD signal, such as heartbeat artifact and the photo bleaching effect, the average response of the blank condition (no stimulation) is subtracted from each stimulated trial. Thus, the imaged signal (Δf/f) reflects the changes in fluorescence relative to the blank trials. For further analysis, VSD signal and maps were computed by averaging over all trials. In the opto-stimulation sessions, due to the laser light artifacts (see Supplementary Figs. S2–S5), the frames of stimulation were removed from analysis (t = 0 and t = 10) as well as any pixels showing bleaching due to the laser light stimulation, i.e., pixels with large negative values around the laser tip, occurring immediately following the laser offset. To compare the microstimulation sessions to optostimulation, we analyzed the VSD data from both types of experiments from t = 20 ms after ICMS onset or optostimulation onset (in the optostimulation data, the 20 ms after laser stimulation onset included the light artifact).

#### Defining region of interests (ROIs)

To study the time course (TC) of the VSD signal in the different sessions, a region of interest (ROI) was defined at the peak response of population activation (the ROI was defined as a circle with a radius = 5 pixels, to fit with all experimental conditions). By averaging the VSD signal over pixels within the ROI we obtained the TC of VSD response for that ROI.

#### Space time analysis (ring ROI analysis)

To quantify and compare the spatial spread of the VSD signal for the different stimulation techniques, we applied a space–time analysis (Fig. 4). We generated a continuous set of non-overlapping circles shaped rings, centered over the peak of the evoked response (supplementary Fig. S7). The size of each ring changed from the center at steps of 50 µm (one pixel) to create a set of 40 consequential rings (covering 2mm radius from the center). The activation for the central ring was defined to be mean response across rings no. 1–5 (to cross a threshold of minimal number of pixels).

#### Map of decay time to threshold

To study the dynamics of the VSD signal after the end of stimulation, we analyzed the response decay time to a threshold amplitude of the VSD signal in the ICMS and opto-stimulation conditions (Fig. 5). To characterize the two phases of the VSD decay signal back to baseline we created two sets to decay time maps for each session by defining a high threshold (70%; for the early fast decay) and a low threshold (30%; for the slower decay) of peak response. Then, we calculated for each pixel, the exact time for the VSD descending transient to arrive below that threshold. We used a linear interpolation on the VSD signal to define the exact time for crossing the threshold, for each pixel (t > 20 ms from stimulation onset). (One session from the ICMS experiments was excluded in this analysis, because it showed outlier decay values; we note that the results were maintained also when including this session).

#### Statistical analysis

To compare the results across different sessions and conditions, we used nonparametric tests: rank-sum or sign-rank. Data are presented as mean ± SEM unless specified otherwise (we computed also median which showed similar values and results). Bonferonni correction was applied for multiple comparisons.

### Supplementary Information

Supplementary Information.

## Data Availability

The datasets generated and analyzed during the current study available from the corresponding author on reasonable request.
