# Supplementary material for: Spatio-temporal activation patterns of neuronal population evoked by optostimulation and the comparison to electrical microstimulation
Source: Sci Rep. 2023 Aug 4;13:12689. doi: 10.1038/s41598-023-39808-w (PMC10403613; doi:10.1038/s41598-023-39808-w)
Supplement: Supplementary file 1 — Supplementary Information. [file 41598_2023_39808_MOESM1_ESM.docx]

**Supplementary material**


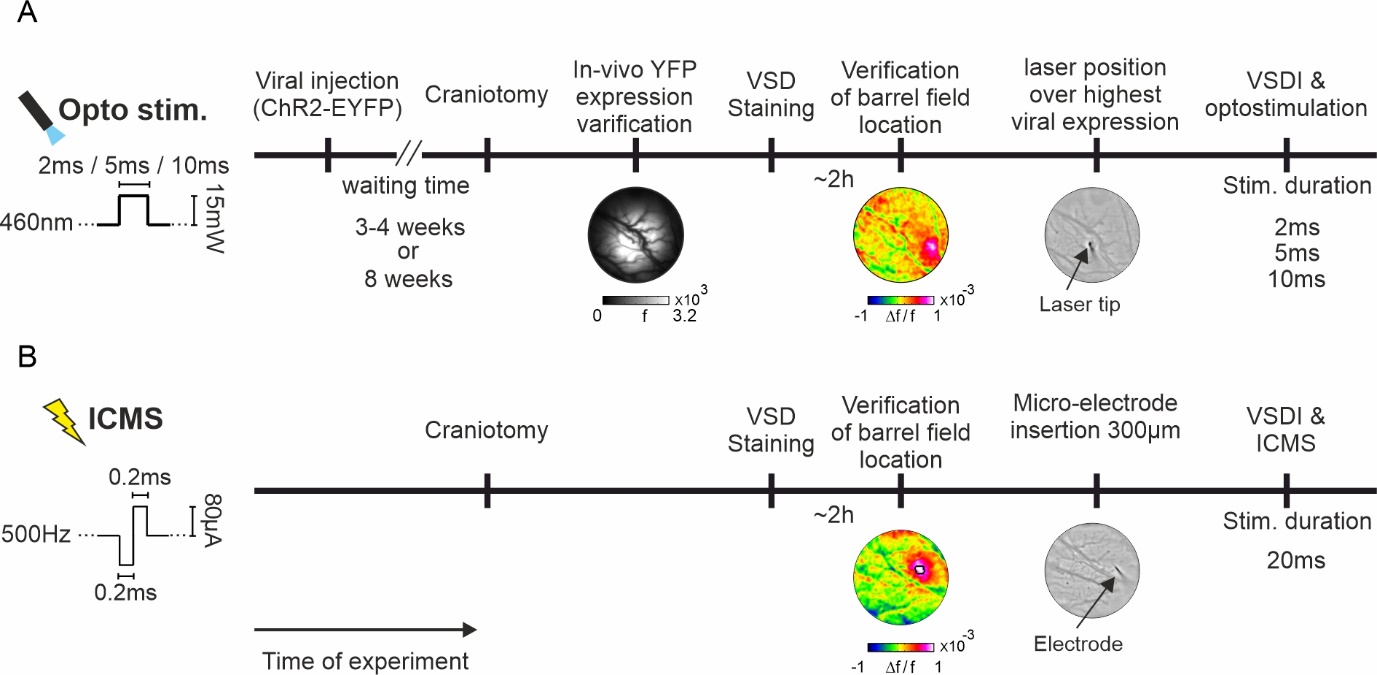


**Supplementary Figure S1: Illustration of the stimulation parameters and experimental timeline. (A)** Left: opto-stimulation parameters: one pulse (2 ms, 5 ms or 10 ms, 460 nm, 15mW) was delivered through a laser fiber. Right: Illustration and timeline of optogenetic experimental design. Below the time line are example maps (from left to right): in-vivo YFP expression, VSD maps of whisker deflection at peak response time and fiber tip position over the cortical site with the highest YFP expression. **(B)** Left: ICMS was delivered through a microelectrode inserted at the upper layers of the barrel field. Microstimulation parameters: train duration: 20 ms (10 biphasic pulses at 500Hz); Each pulse was comprised of a cathodal pulse followed by an anodal pulse (0.2ms each). Current amplitude: 80µA. Right: Illustration and timeline of ICMS experimental design. Below the timeline are example maps (from left to right): VSD map for whisker deflection at peak response time and an image of the blood vessels patterns with the microelectrode position in the barrel cortex.

**Laser artifacts when combining opto-stimulation and VSDI**

Our first goal was to demonstrate that we are able to combine optostimulation in the cortex using a laser fiber at 460 nm, with the VSDI at 630 nm wavelength for VSD excitation. Because both techniques are based on optical signals, we attempted first to investigate possible laser light artifact during VSDI. To evaluate this, we first directed the stimulating laser fiber to a naïve rat barrel cortex, without VSD staining (Supplementary Fig. S2) or with VSD staining (Supplementary Fig. S3,4). In both conditions, we found that the laser pulse generated an optical artifact, centered around the fiber's tip during stimulation, that showed increasing amplitude with the laser power. This part of the artifact indicates that the laser light was reflected back from cortical surface, passing through the VSDI dichroic and emission filters and arriving at the camera’s sensor. When further investigating the influence of the laser stimulation with the VSD staining (Supplementary Fig. S3 and S4 for 10ms pulse of 100ms pulse respectively) we observed a negative VSD signal around the fiber's tip immediately following stimulation offset. This result was previously reported in Lim et al. (Lim et al., 2012) and was suggested to originate from a photo bleaching of the VSD, due to the light stimulation of the VSD. Both the positive and negative parts of this artifact increased with laser power (Supplementary Fig. S3B,4B inset) and the max and min signal amplitude varied linearly with the laser's power (Supplementary Fig. S3C, 4C). Previous studies demonstrated that cortical circuit activity controlled by ChR2 (excitation at 460/470 nm) can be imaged with VSDI, simultaneously (VSD excitation at ~630nm). The two wavelength excitations are sufficiently separated, thus enabling an integration of the two methods (Airan et al., 2007; Matyas et al., 2010; Mohajerani et al., 2013).

Next, to determine if the laser stimulation (at the used experimental parameters) interferes with the neuronal population responses, we delivered a laser pulse (10ms) at 15mW simultaneously with a single whisker deflection (1pulse, 50ms duration) to a WT rat barrel cortex (no viral injection). Supplementary Fig. 5 shows the separate VSD responses to laser stimulation alone, whisker stimulation alone and their combination. Next, we compared the population response after stimulation offset (to avoid the artifact), and found that the VSD response to the laser stimulation combined with whisker deflection is similar to the linear sum of the VSD responses to the whisker deflection alone and laser stimulation alone (Supplementary Fig. 5C). Moreover, the VSD response to the whisker is similar to that of the laser + whisker condition after subtraction of the laser alone effect (Supplementary Fig. 5D), suggesting a linear relation between the laser artifact and the VSD response evoked by a whisker stimulation. In summary, the laser light stimulation is reflected back from cortical surface, causing a light artifact in the camera and inducing a transient photo-bleaching effect at the vicinity of the fiber's tip. Therefore, to avoid the effects of the laser artifacts, our analysis will focus on the neuronal responses measured after light stimulation offset and we remove pixels with photo-bleaching from data analysis. In addition, we showed that despite the laser artifacts, neuronal response can be detected reliably after optostimulation offset and the population response evoked by whisker deflection – is unchanged, following the laser stimulation in a naïve animal.


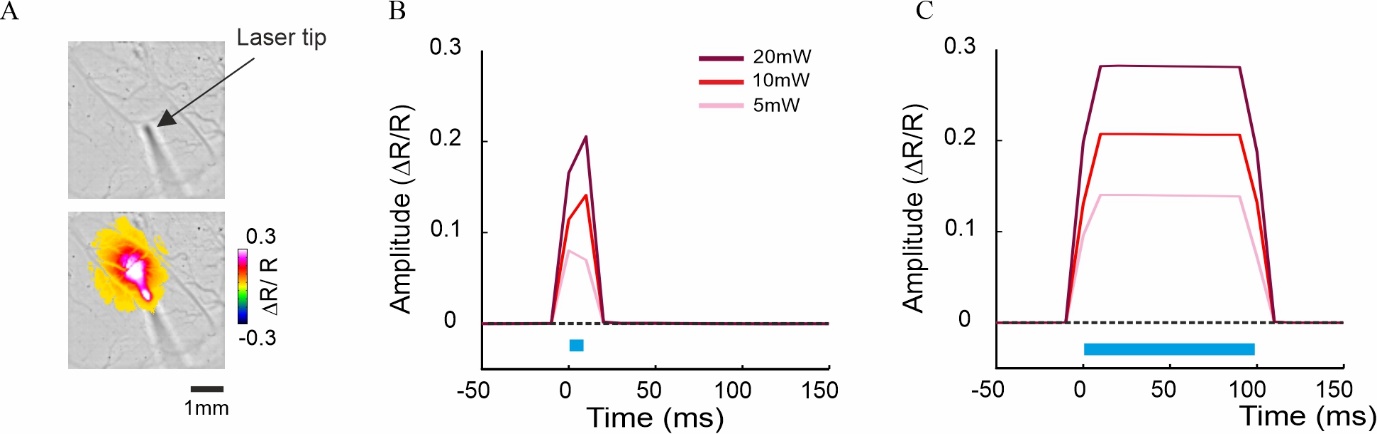


**Supplementary Figure S2: Control experiments of the laser fiber on non-stained barrel cortex in the VSDI setup. (A)** A reflection map (∆R/R) of the imaged cortex at 10ms post stimulation onset (10ms pulse width). The map is color coded. Barrel cortex was illuminated with 630 nm. **(B,C)**: Time course of the ∆R/R signal in a circle ROI around the laser tip of 10ms (B) or 100ms (C) laser stimulation. Three different laser power were used (5mW, 10mW and 20mW). Blue bars depict the light stimulation duration.


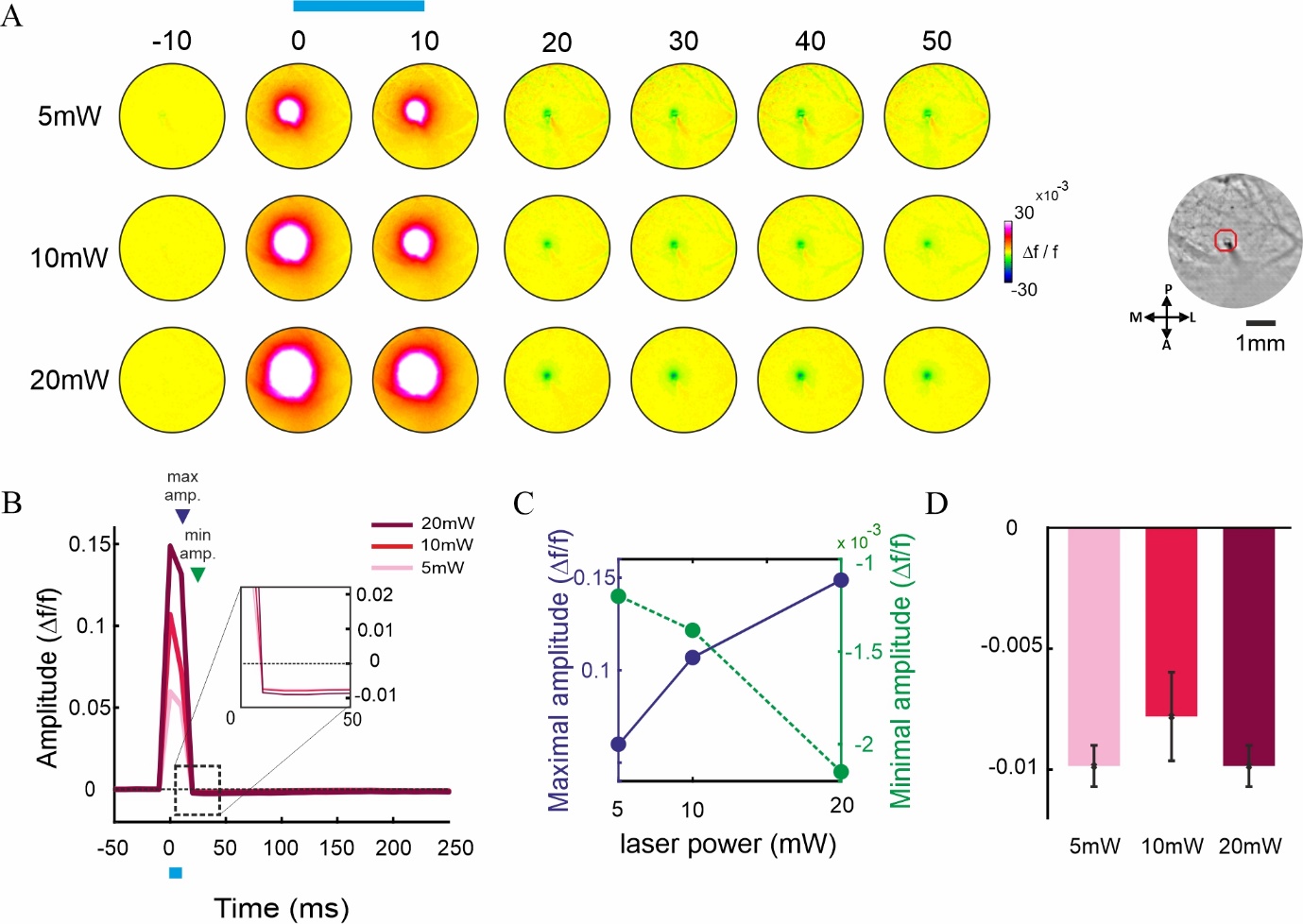


**Supplementary Figure S3: The effect of 10ms fiber laser stimulation in WT rats barrel cortex after VSD staining.** (**A)** Left: maps of VSD response following optostimulation in the barrel cortex of a WT rat (i.e. not injected with a virus). The numbers above the maps represent the time in ms after the optostimulation onset. Right: the position of the laser fiber over the blood vessels image. The red circle ROI is centered on the peak response of the VSD map. Maps are color coded (Δf/f). **(B)** Time course of the imaged signal at a ROI around the fiber's tip. Inset: an enlargement of the declining phase. Blue bar depicts the stimulation duration. **(C)** Maximal and minimal response (same ROI in B) as function of laser power. **(D)** Amplitude of the negative response normalized to peak response for each stimulation power.


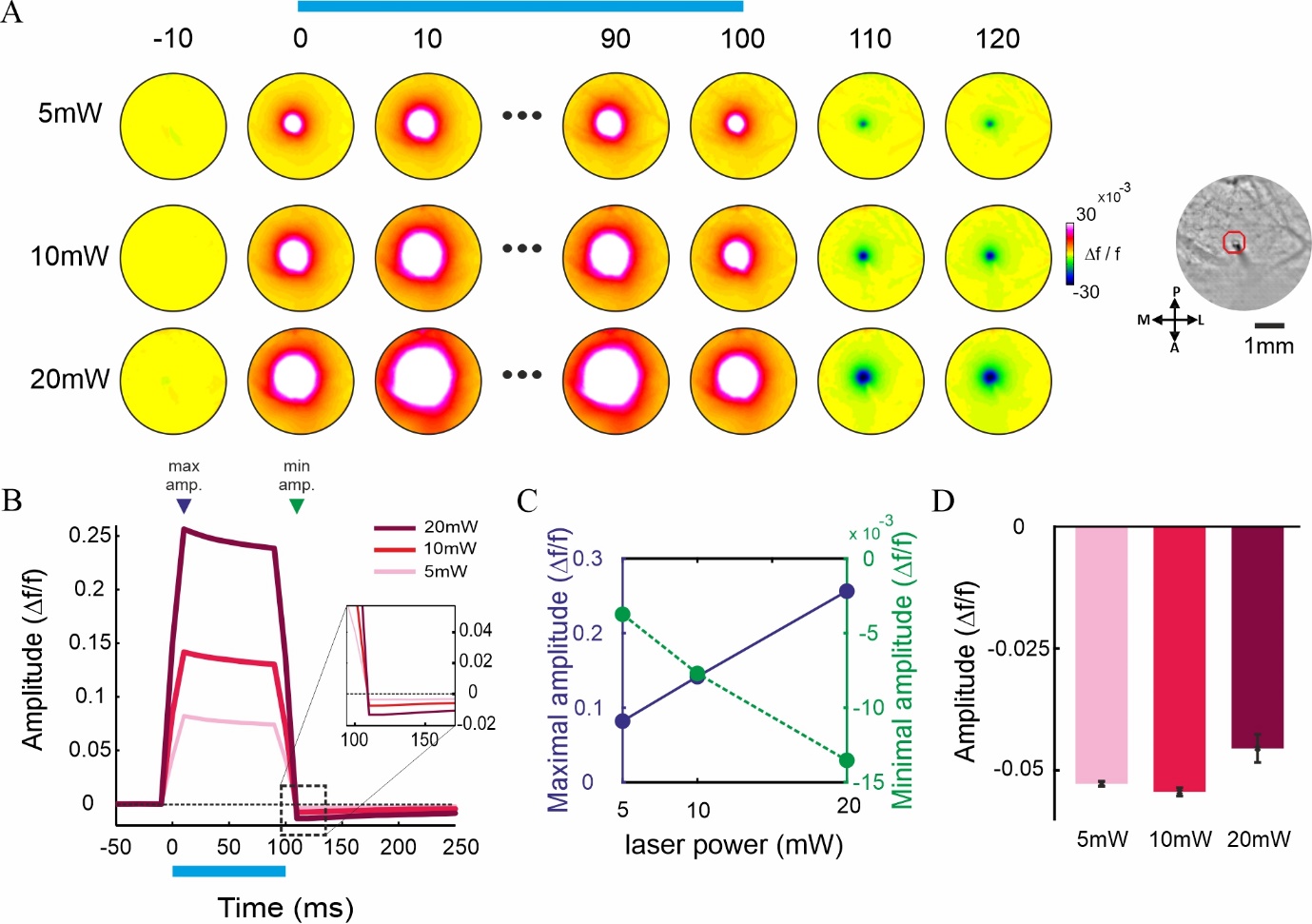


**Supplementary Figure S4: The effect of 100ms fiber laser stimulation in WT rats barrel cortex after VSD staining.** Same as Supplementary Figure S3 but for longer optostimulation of 100ms.


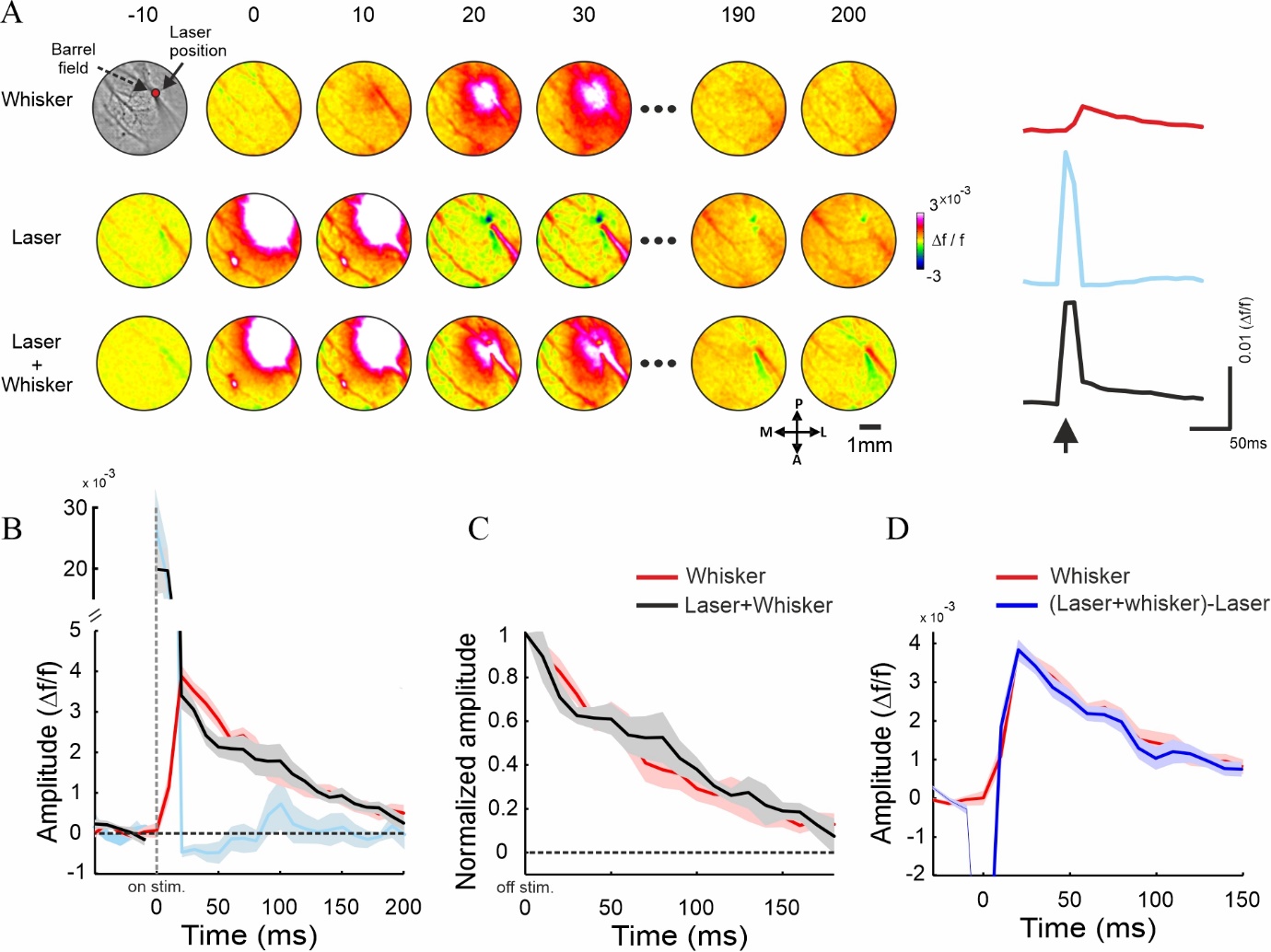


**Supplementary Figure S5: Whisker deflection combined with laser stimulation in a WT rat, a VSD control experiment.** **(A)** Left: Population response maps evoked by whisker C2 deflection (top), laser stimulation (middle) and the combination of laser stimulation and whisker deflection (bottom) in the barrel cortex of a WT rat (not injected) after staining the cortex with VSD. The numbers above the maps represent the time in ms after the stimulation onset. ROI denotes the barrel field of whisker C2. Maps are color coded (Δf/f). Right: time course of the VSD signal at the barrel field ROI. **(B)** The time course of the VSD signal in the ROI following whisker deflection (red), laser stimulation (blue) and laser + whisker (black). Time=0 is stimulation onset. The shaded area represents ±1SEM over trials (n=10 trials for each stimulation condition). **(C)** The normalized time course aligned on stimulation offset (time = 0). Shaded area represents ±1SEM over trials. **(D)** Time course of the VSD signal following deflection of C2 whisker compared to (laser + whisker) minus (laser stimulation alone). Shaded area represents ±1SEM over trials.

**Quantification of the YFP expression**

At the beginning of each experiment and before staining the cortex with VSD for imaging, we measured the in-vivo ChR2-YFP expression in the barrel cortex. YFP fluorescence images were taken in the Short_Exp_ and Long_Exp_ animals and then we performed the following analysis. First, the YFP image were normalized between 0-1, to account for the different illumination conditions in which the fluorescence image was performed in the different animals. Then, we computed the number of pixels crossing a threshold of 2STDs from mean fluorescence value of the YFP map. Supplementary Figure S6A shows two example maps, where the colored pixels marks YFP pixels crossing 2STDs above the mean value. The LongExp map shows a higher pixel count than the ShortExp. This is further quantified in Fig R4B for all animals, and shows that the number of pixels was larger in the Long_Exp_ vs. Short_Exp_: Long_Exp_, 438 ± 52.9, Short_Exp_, 231.7±92. While the difference between the groups is not-significant (206 pixels; each pixel is 50^2^µm^2^), the value for shuffled data (mean over all random permutation) is 21±131, which is 1.5 STD lower than the real data (Fig. S6B, inset). In summary, these results indicate that the YFP expression showed a trend toward larger values in the Long_Exp_.

We note that Long_Exp_ showed also a larger spatial spread of VSD activity (see Fig. 4) – which agrees well with a larger YFP expression. To study the relation between the VSD response amplitude and YFP expression value, we selected an ROI at the peak VSD response map (same as in Fig. 3) and calculated the YFP expression (in STD units) averaged over the ROI's pixels (Fig. S6Ci). The mean YFP value was smaller in the Short_Exp_ vs. the Long_Exp_ – however, this was n.s. Next, we computed the VSD response in the ROI, as shown in Figure S6Cii. The VSD response was higher for the Long_Exp_ relative to the Short_Exp_ condition (Short_Exp_, 0.17x10^-3^±0.4x10^-3^; Long_Exp_, 3.6 x10^-3^±0.4 x10^-3^; p< 0.05).


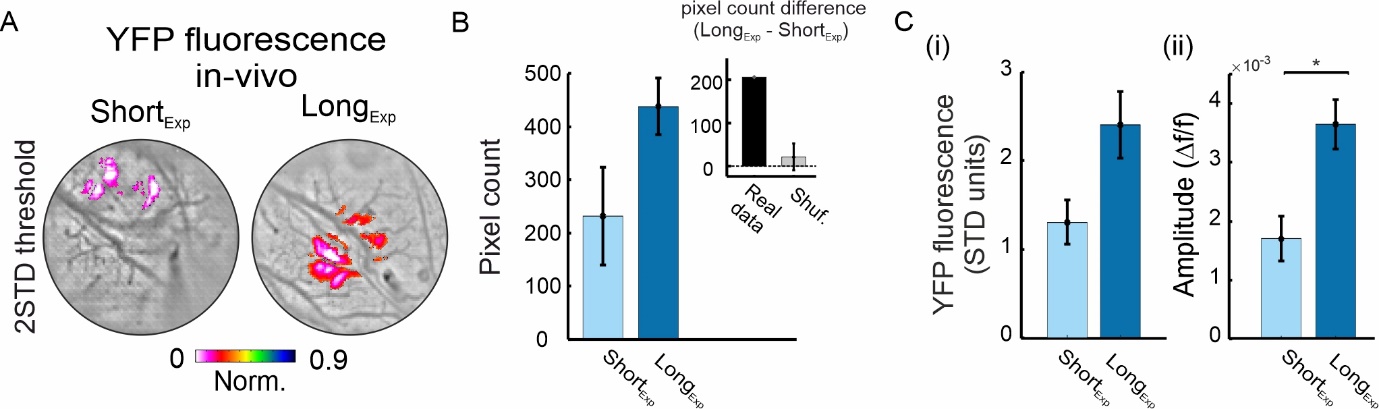


**Supplementary Figure S6: Quantification of the YFP image in the Long and Short expression periods.** **(A)** YFP pixels crossing a threshold of 2STD (from mean fluorescence of YFP image) superimposed over blood the vessel map, for an example session of Short_Exp_ (left) and Long_Exp_ (right). **(B)** The number of pixels crossing a threshold of 2STDs from the mean YFP fluorescence (Short_Exp_, n=3; Long_Exp_, n=3). Numbers are mean±SEM. Inset: the pixel count difference between Long_Exp_ and Short_Exp_ in the real data (black bar) and in shuffle data (gray bar; mean over all random permutations, error bar: ±1SEM). Each pixel is 50^2^µm^2^. **(C( i:** YFP florescence (in STD units) averaged over the pixels of a circle ROI centered at peak VSD response (same ROIs as in Fig. 3; Short_Exp_, n=5; Long_Exp_, n=7 sessions). **ii**: VSD amplitude at peak response (20 ms post stimulus onset), same ROIs as in (i), * <0.05, Wilcoxon rank-sum test.


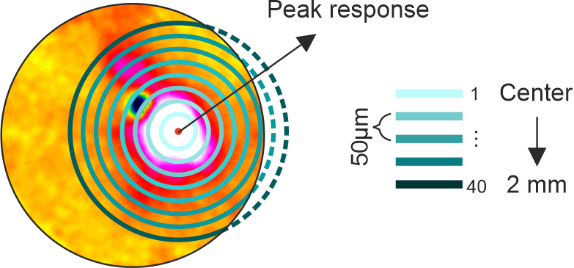


**Supplementary Figure S7**: **Illustration for the ring analysis in Fig. 4**. An illustration of circle shaped rings ROIs centered over the peak of the evoked VSD response (red dot). We generated a set of 40 consecutive non-overlapping rings, increasing in radius from the center (light blue) at steps of 50 µm (one pixel) up to 2 mm radius from the center (dark blue).
